# Expression of cancer-testis antigen in patients with non-small cell lung cancer and its clinical relevance

**DOI:** 10.1186/2051-1426-1-S1-P246

**Published:** 2013-11-07

**Authors:** Xin-Feng Chen, Meng Wang, Dong-Li Yue, Jin-Yan Liu, Lan Huang, Feng Li, Song Zhao, Yu Qi, Wei Hu, Xiang-Nan Li, Yi Zhang

**Affiliations:** 1 Biotherapy Center, the First Affiliated Hospital of Zhengzhou University, Zhengzhou, China; 2 Department of Oncology, the First Affiliated Hospital of Zhengzhou University, Zhengzhou, China; 3 Department of Thoracic Surgery, the First Affiliated Hospital of Zhengzhou University, Zhengzhou, China; 4 Department of Biological Engineering, Zhengzhou University, Zhengzhou, China

## Background

Lung cancer is the most common cause of cancer-related mortality worldwide. Survival rates after non-small cell lung cancer (NSCLC) diagnosis still remain poor, despite standardization of surgery and adjuvant treatment. Cancer testis (CT) antigens as targets for immunotherapy in cancer patients have been heavily investigated. We aim to evaluate the expression of CT antigens in non-small cell lung cancer and the correlation with the clinical characteristics.

## Methods

The expression of MAGE-A3, MAGE-A4, MAGE-C2 and NY-ESO-1 in fresh cancer tissues from 113 patients with non-small cell lung cancer were analyzed by RT-PCR. The expression of HLA-A2 was detected by flow cytometry.

## Results

49.6% non-small cell lung cancer samples were HLA-A2 positive. CT antigens were frequently expressed in non-small cell lung cancer. The expression percentage of each gene in cancer tissues samples were as follows: MAGE-A3, 70.8%; MAGE-A4, 46.9%; MAGE-C2, 61.1%; and NY-ESO-1, 15.0%. In all, Seventeen tissues did not express any of the CT antigens tested, 85.0% expressed at least one, 66.4% co-expressed two, 34.5% co-expressed three, 6.2% co-expressed four examined CT antigens. The percentage of samples co-expression HLA-A2 and CT genes were: MAGE-A3, 51.6%; MAGE-A4, 36.3%; MAGE-C2, 44.0%; and NY-ESO-1, 12.1%. MAGE-A3 was associated with male, smoking history, tumor stage, tumor differentiation and lymph node metastasis (P<0.05); MAGE-C2 was associated with tumor stage (P<0.05). There was no correlation MAGE-A4 or NY-ESO-1 expression with clinical characteristics such as gender, age, HLA-A2 positive, clinical stage, grade of differentiation and lymph node metastasis (P>0.05).

## Conclusion

CT antigens might be prognostic markers and factors related to the progress of NSCLC, and also be served as the immunotherapeutic targets of NSCLC, especially in multi-antigen vaccine preparations and tumor antigen specific lymphocytes infusion.

